# Fungal ADP-ribosylation: an emerging signalling pathway in adaptation and virulence

**DOI:** 10.1042/BST20260689

**Published:** 2026-10-29

**Authors:** Johanna Mary Martha Gosciniak, Johannes Gregor Matthias Rack

**Affiliations:** Medical Research Council Centre for Medical Mycology, University of Exeter, Department of Bioscience, Faculty of Health and Life Sciences, Geoffrey Pope Building, Stocker Road, Exeter, EX4 4QD, U.K.

**Keywords:** medical mycology, oxidative stress, PARG, PARP

## Abstract

ADP-ribosylation is a posttranslational modification conserved across all domains of life and some viruses. While it is well established as a critical signalling mechanism in humans and is widely exploited as a therapeutic target in cancer, its roles in other kingdoms, particularly *Fungi*, remain poorly understood. Despite this, recent advances highlight the importance of fungal ADP-ribosylation in the regulation of key cellular maintenance processes such as DNA damage and oxidative stress responses, morphogenesis, growth, and proliferation. Here, we summarise our current understanding of fungal ADP-ribosylation and how it supports stress adaptation and virulence. While many aspects of fungal ADP-ribosylation remain unexplored, emerging discoveries underscore its importance in fungal biology and hint at a great potential for future antifungal therapies.

## Introduction

Posttranslational modifications (PTMs) are a diverse set of covalent chemical alterations of protein backbones or side chains and can be either additive (e.g. acetylation, phosphorylation, or ubiquitination), subtractive (e.g. proteolysis or dephosphorylation), or transformative (e.g. citrullination) [1,2]. In *Eukarya*, they expand the genomically encoded repertoire from tens of thousands to potentially millions of functional protein variants, allowing the dynamic and intricate regulation of biological activities [1,2].

Among the over 650 PTMs known today, ADP-ribosylation stands out because of its diverse attachment sites, including acidic (Glu/Asp), basic (Arg/Lys), hydroxy (Ser/Tyr), and thiol (Cys) functionalities; its ability to modify non-protein targets, including nucleic acids and small molecules; and its occurrence as either monomeric (termed MARylation) or polymeric (PARylation) modification [3–5]. The ADP-ribosylation reaction involves the transfer of an ADP-ribosyl moiety from the metabolic cofactor NAD^+^ onto an attachment site by an (ADP-ribosyl)transferase (ART). Most ADP-ribosylation is carried out by members of two related superfamilies: the PARPs, also known as diphtheria toxin-like ARTs, which are characterised by a catalytic H-Y-[EDQ] motif, and the cholera toxin-like ARTs (ARTCs), which carry an R-S-E motif [6,7]. Both PARPs and ARTCs are evolutionarily ancient and can be found in all kingdoms of life as well as some viruses. Some ARTs of the PARP superfamily can further extend the initial MARylation into linear or branched PARylation (Figure 1). PARylation was first described in the mid-1960s as a reaction occurring in mammalian nuclear extracts [8–10]. Over the following decades, ADP-ribosylation signalling has been established as not only a key regulator of the DNA damage response (DDR) but also an important contributor to a multitude of other cellular processes, including response to abiotic stresses, cell proliferation, growth, morphology, and telomere maintenance [4,5,11]. Manipulation of ADP-ribosylation signalling has found successful application in cancer therapy, for example, as first-line maintenance therapy for advanced ovarian cancer, and it is also being explored for the treatment of other diseases driven by oxidative stress, DNA damage, or inflammatory signalling, such as neurodegenerative, viral, and metabolic diseases [5,12,13]. Despite these successes, our understanding of ADP-ribosylation in many other important branches of life remains limited. The present review explores recent advances in our understanding of fungal ADP-ribosylation signalling, its emerging roles in (patho)physiology, and the potential of targeting these pathways as novel antifungal therapy. While first insights suggest the general importance of ADP-ribosylation systems in fungi, we will primarily focus on fungal PARP1 as the best studied example.

**Figure 1:**
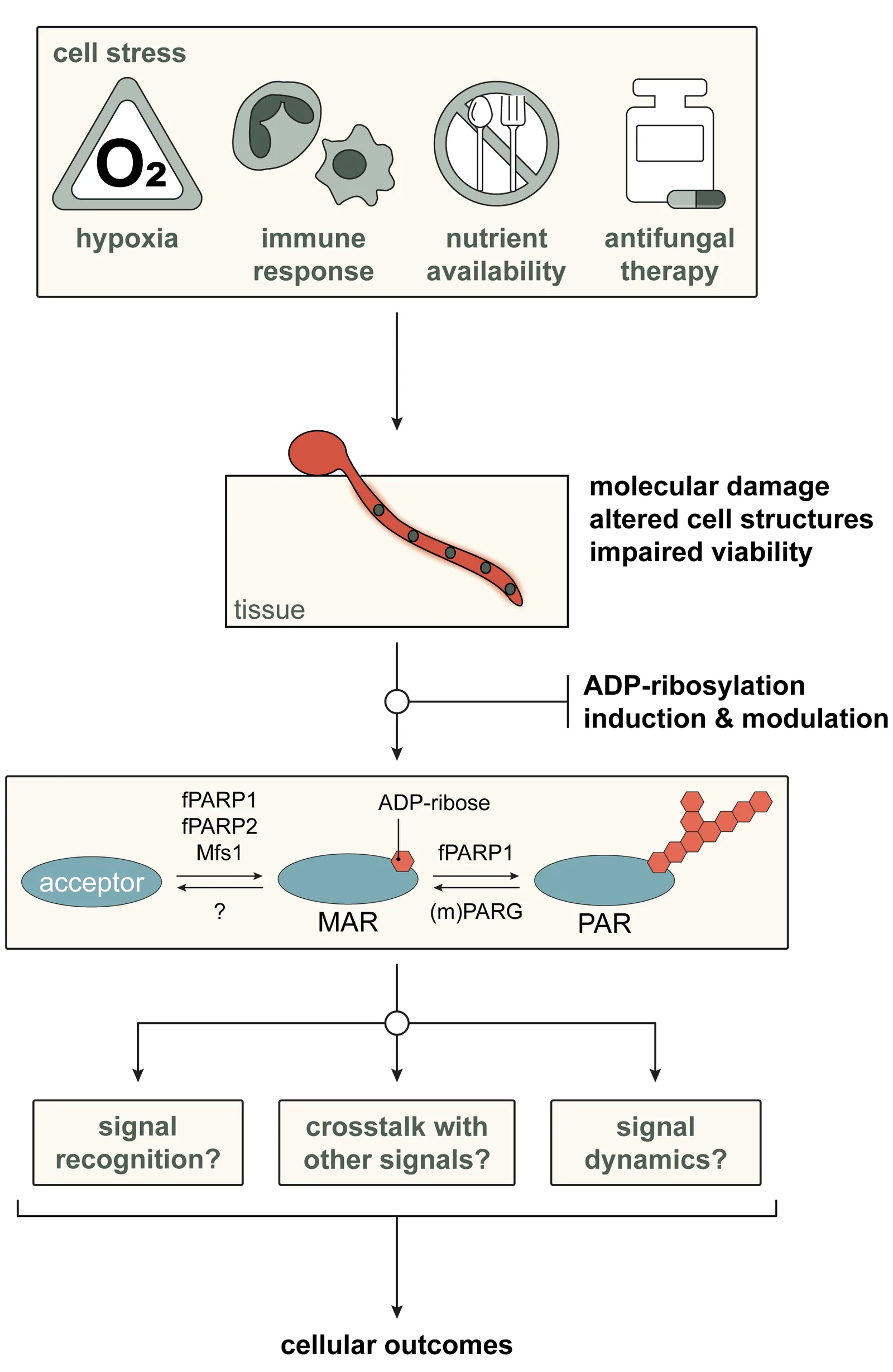
Proposed link between fungal stress responses and ADP-ribosylation signalling Endogenous and exogenous sources of stress can damage cellular macromolecules (e.g. through oxidation or alkylation), impairing their function. This can also alter cellular structures and impair fungal viability. To adapt and overcome these stresses, fungi mount an adaptive response including ADP-ribosylation. Signal establishment is distinct, with fungal PARP2 (fPARP2) and macrodomain-fused SirTM 1 (Mfs1) only being able to create MARylation, while fungal PARP1 (fPARP1) can extend the initial modification, forming a PARylation signal. A major trigger for fPARP1 activation is DNA damage; however, whether other triggers exist, how fPARP2 is activated, and whether signalling, like in humans, requires further modulators remains to be established. Moreover, how ADP-ribosylation is recognised and interpreted, if the ADP-ribosylation pathways cross-talk with other pathways, and how establishment and removal dynamics influence cellular outcomes remain to be determined.

## Why care about *Fungi*?

*Fungi* represent a diverse, yet historically understudied kingdom comprising an estimated 2–3 million species, of which only about 155,000 have been described [14]. Fungi form complex relationships with their environment and other species therein. These interactions range from indirect, as exemplified by saprotrophs, which break down dead biological material and recycle nutrients back into the ecosystem, to obligate dependencies that can be symbiotic, parasitic, or pathogenic in nature [15,16].

Reflective of their diverse nature, fungi have been harnessed for biotechnology and food production but can also cause diseases in plants and animals, resulting in devastating loss of crops and substantial mortality worldwide [17–20]. An estimated 2.55 million deaths are directly attributed to fungal infection annually, 95.8% of which are caused by just four genera: *Aspergillus, Candida, Cryptococcus*, and *Pneumocystis* [17,21]. The World Health Organisation recognised fungal pathogens as critical to public health in 2022, citing challenges in diagnosis and treatment together with the increasing emergence of antifungal resistance, and published the first fungal pathogen priority list [22]. One of the defining features amplifying this threat is the ability of fungal pathogens to rapidly adapt and evolve under new environmental pressures. In the host niche, these pressures are typically derived from a combination of endogenous and exogenous sources (Figure 1), including elevated temperature, hypoxia, increased CO_2_ concentration, nutrient availability, and trace metal starvation, as well as external factors such as antifungal treatment [17,23].

## What is the fungal repertoire of ADP-ribosylation signalling enzymes?

Like most PTMs, ADP-ribosylation is transient: it is written, read, interpreted, and ultimately erased [3,5,24]. Several evolutionarily unrelated protein families possess the ability to utilise NAD^+^ in an ADP-ribosyl transfer reaction, including PARPs, ARTCs, sirtuins, and Toll/interleukin-1 receptor domains, and they are collectively termed ARTs [3,5,6,25–27]. While phylogenetic analyses suggest that additional fungal ARTs may exist [6], only four ADP-ribosylation writer enzymes have been described to date: two PARPs, the related tRNA 2′-phosphotransferase 1, and a ‘class M’ sirtuin (termed macrodomain-fused SirTM 1 [Mfs1]) [27–31].

The PARP superfamily can be further subdivided into six clades, with fungal proteins forming distinct branches within clades 1 (fPARP1) and 6 (fPARP2) (Figure 2) [32,33]. Clade 1 proteins are among the best characterised and include the DNA-damage-responsive human PARP1–3, whereas relatively little is known about clade 6 enzymes, which include human PARP6/8/16. Phylogenetic analyses suggest that both fPARP1 and 2 were present in the last common ancestor of *Fungi* but have undergone several independent loss events [32]: fPARP1 enzymes are common among the early divergent fungi, *Basidiomycota*, and *Pezizomycetes* (part of the *Ascomycota* division), but are nearly absent in yeasts such as *Saccharomycotina* and *Schizosaccharomycetes*, suggesting an association with species possessing multicellular hyphae and/or more elaborate developmental programmes [11,28,30,32]. Their presence has been experimentally confirmed in several fungal species, including *Aspergillus* species (in *A. nidulans* and *A. fumigatus), Batrachochytrium dendrobatidis, Fusarium oxysporum* f. sp. *niveum, Magnaporthe oryzae, Neurospora crassa, Podospora anserina*, and *Trichoderma guizhouense* [28–30,34–37]. Less is known about the distribution of fPARP2 except that it is present in some agriculturally and medically important classes of *Ascomycota*, including *Eurotiomycetes* and *Sordariomycetes*, as well as in some *Basidiomycota* [32].

**Figure 2:**
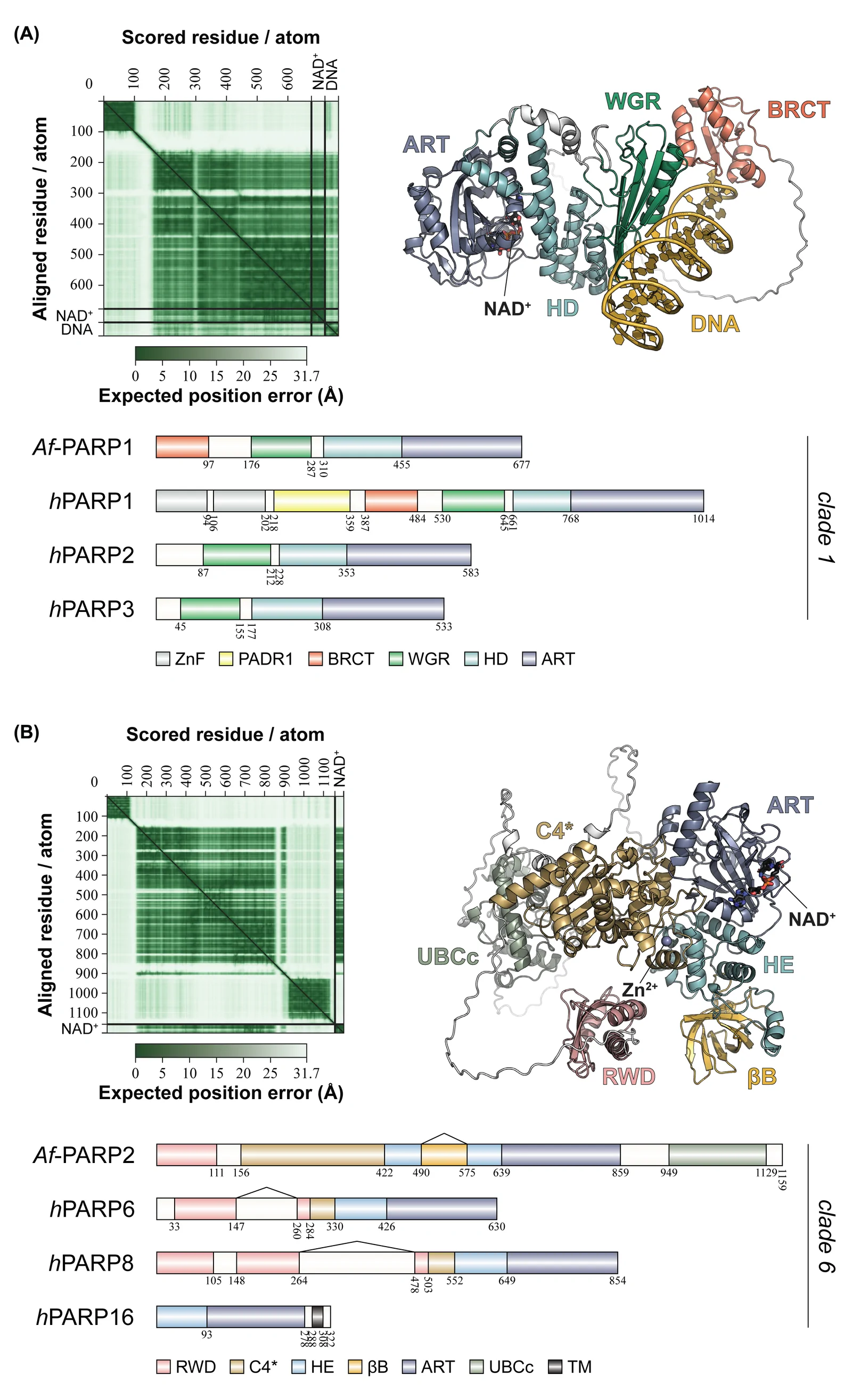
Domain architecture of fungal PARPs (**A**) Domain architectures of clade 1 PARPs from *A. fumigatus* (as an example for fPARP1s) and humans. Top panels show an AlphaFold3 model and associated predicted aligned error (PAE) scores of *A. fumigatus* PARP1 (*Af*-PARP1) in the presence of NAD^+^ and a nicked DNA mimetic. The model has global pTM of 0.64 and an ipTM of 0.69. Lower panel shows a schematic representation of the domain organisation of the selected proteins with domain or motif names given below. Disordered regions are shown as white. Residue numbers corresponding to approximate domain or motif boundaries are indicated below. (**B**) Domain architectures of clade 6 PARPs from *A. fumigatus* (as an example for fPARP2s) and humans. Top panels show an AlphaFold3 model and associated PAE scores of *Af*-PARP2 in the presence of NAD^+^ and zinc. The model has global pTM of 0.61 and an ipTM of 0.96. Lower panel shows a schematic representation of the domain organisation of the selected proteins with domain or motif names given below. Split domains are indicated by connecting lines above the scheme. Abbreviations: 4C*, PARP6/8-type C4 zinc finger; βB, beta-barrel; BRCT, Breast cancer type 1 susceptibility protein (BRCA1) C-terminal domain; HD, alpha-helical regulatory domain; HE, helical extension; PADR1, poly(ADP-ribosyl)polymerase-associated domain 1 (also known as the third zinc finger domain [ZnF3]); RWD, after the three major RWD-containing proteins: RING finger-containing proteins, WD-repeat containing proteins, and yeast DEAD (DEXD)-like helicases; TM, transmembrane domain; UBCc, ubiquitin-conjugating enzyme E2 catalytic domain; WGR, after conserved Trp-Gly-Arg motif; ZnF, PARP-type zinc fingers.

The domain architecture of fPARP1 largely conforms to that of the other clade 1 enzymes (Figure 2A). Specifically, they contain the DNA recognition domain WGR (named after the conserved Trp-Gly-Arg motif), an autoregulatory HD domain as well as the catalytic ART domain. However, in contrast with human PARP1 (*h*PARP1), they do not contain the N-terminal zinc fingers, but, unlike *h*PARP2/3, maintain an N-terminal BRCT (BRCA1 C-terminus) domain.

Like fPARP1, fPARP2 is a multidomain protein. Initial sequence analysis suggested the high-confidence presence of an ART domain and a C-terminal ubiquitin-conjugating enzyme E2 catalytic domain, the latter being a distinguishing feature of fPARP2 compared with its mammalian orthologues. In addition, two low-confidence Pfam-B domains were identified. These included a fungal-specific N-terminal region, termed the fungal PARP E2-associated domain, which shares sequence similarities with RWD domains (Pfam-B_30617), and a domain of unknown function (Pfam-B_2311) [32]. However, more recent AlphaFold-assisted analyses replaced the PfamB domains and suggest that fPARP2 has a domain organisation similar to that of mammalian PARP6/8 (Figure 2B) [28,38]. In addition to the ART and E2 domains, fPARP2 contains four other domains: (i) an RWD domain, a proposed E2-interacting module named after the main protein families with which it is associated: RING finger-containing proteins, WD repeat-containing proteins, and DEAD-like helicases; (ii) an uncharacterised 4C*-type zinc-binding domain; (iii) a helical extension (HE) domain suggested to have regulatory function similar to the HD domain of *h*PARP1–3 and fPARP1; and (iv) a fungal-specific β-barrel insertion in the HE domain of unknown function. However, experimental validation of the precise domain architecture and functional roles of these domains remains outstanding.

ADP-ribosylation reversal is carried out by enzymes from one of the three evolutionarily unrelated superfamilies of ‘erasers’: macrodomains, (ADP-ribosyl)hydrolases (ARHs), and NADARs (‘*NAD*- and *A*DP-*r*ibose’-associated enzymes) [39–41]. Little is known about these enzymes in *Fungi*: to date, no NADAR-type enzyme has been identified, and the evidence for fungal ARHs and macrodomains is sparse [42–44]. Phylogenetic analyses suggest the presence of four macrodomain classes (ALC1-like, Macro2-type, MacroD-type, and PARG-like) [29,39], of which Macro2, MacroD, and mPARG have some experimental support as potential ‘erasers’ or have related activities [42,44–46]. Notably, PARG-like enzymes are classified into two major subtypes: canonical PARGs, found in mammals, and microbial PARGs (mPARGs; formerly DUF2263). The apparent presence of both subtypes within the fungal kingdom raises intriguing questions regarding the acquisition, evolution, and potential functional diversification within *Fungi*. The distribution of the Macro2-type class (InterPro IPR043472) appears to be limited to fungal and some bacterial species [39]. Similarly, ARH-type erasers are predicted to be present in *Fungi* and have been shown to possess ARH activity *in vitro* [43], although little else is currently known about their biological function.

While there is good evidence for signal establishment and removal of enzymes in *Fungi*, it remains an open question how the ADP-ribosylation signal is ‘read’ and ‘interpreted’. Studies in the mammalian system have identified three major structural domain types able to engage with the modification: WWE, PBZ, and macrodomains. However, so far, no WWE or PBZ domains have been identified in *Fungi*, and while macrodomains are present, most characterised readers belong to the MacroH2A-like class, which has no fungal orthologues [39]. This suggests that either some of the eraser enzymes have undergone fungal-specific adaptation, enabling them to function as readers rather than erasers, or that ADP-ribosylation is recognised through a less modification-specific interaction domain or binding motif, such as the oligonucleotide/oligosaccharide-binding fold domain, forkhead-associated domain, BRCT domain, PilT N-terminal domain, or PAR-binding module, among others [47–52].

## How is the ADP-ribosylation signal established?

The PARP superfamily is characterised by a conserved catalytic H-Y-[EDQ] (polyARTs) or H-Y-[ILY] (monoARTs) triad involved in the formation of the nicotinamide binding pocket [53]. Initially, the H-Y-[EDQ] motif was thought to be the sole indicator for PARylation activity, as Glu>Gln substitution in *h*PARP1 (E988Q) causes a polymer extension deficiency while maintaining MARylation activity [54]. However, *h*PARP3 contains the classical H-Y-E triad yet catalyses only MARylation [53]. Later investigations found a second structural feature of the NAD^+^ binding pocket, the so-called donor loop (D-loop), which is more extended and rigid in *h*PARP1/2 than in *h*PARP3 [53]. This opens up the active site and potentially allows access of an ADP-ribose moiety to the growing PAR chain [53,55].

It has been proposed that the ADP-ribosyl transfer reaction follows an S_N_2 mechanism and processes via the formation of an oxocarbenium intermediate. The NAD^+^ ligand is positioned into an internally strained 3′-*endo* conformation, which closely resembles the expected transition state geometry [56]. This weakens the nicotinamide-ribose bond and helps to drive the reaction. In glutamate-containing PARPs, the bond is further polarised through interaction of the glutamate residue with the 2′′-hydroxyl group of the distal ribose. Moreover, the glutamate is believed to stabilise the oxocarbenium intermediate and, during chain elongation, to increase the nucleophilicity of the acceptor, thereby promoting the formation of the ribose–ribose bond. Conversely, the absence of this glutamate residue in clade 6 PARPs, such as fPARP2, is thought to limit these enzymes to MARylation [53]. While the S_N_2 reaction can produce steric mixtures, conformational restraints within the active site force an inversion at the anomeric carbon of the distal ribose, resulting in conversion of β-NAD^+^ to an α-linked ADP-ribosylation product [56,57]. Our recent investigations of *Af*-PARP1 demonstrated that it is a PARylation writer containing both the canonical H-Y-E triad and an *h*PARP1/2-like D-loop [28]. These observations support the idea that animal and fungal PARPs utilise a common mechanism. Noteworthy, animals carry an auxiliary protein, termed histone PARylation factor 1 (HPF1), which interacts with PARP1/2, forming a composite active site [58,59]. This introduces a second catalytic glutamate residue and dramatically alters substrate specificity from glutamate/aspartate in the absence of HPF1 to serine in its presence [59,60]. Earlier analyses suggest, however, that HPF1 is absent in *Fungi* and emerged in the early evolution of the *Animalia* kingdom [28,61].

In contrast with PARPs, relatively little is known about the catalytic mechanism of SirTM-type ARTs. Initial characterisation of the bacterial SirTM system highlighted two key features: (i) a strict dependence of the target protein to carry another PTM, lipoylation, on a lysine residue structurally adjacent to the ADP-ribose acceptor site, and (ii) alteration of the catalytic HG motif to QG [27]. In the canonical sirtuin deacetylation reaction, NAD^+^ reacts with the acylated lysine, forming an α-1′′-*O*-alkylamidate intermediate. The histidine of the HG motif then acts as a general base to facilitate the intramolecular nucleophilic attack of the 2′′-hydroxyl onto the positively charged iminium carbon, thus allowing formation of a bicyclic intermediate and ultimately release of the demodified lysine [62–64]. Since in SirTMs the deprotonation reaction cannot occur, it appears likely that the *O*-alkylamidate intermediate reacts with a nucleophile in spatial proximity, thus transferring the ADP-ribosyl moiety and leaving the original lysine-alkyl modification unaltered. However, further studies are needed to elucidate the exact reaction mechanism.

## What are the fungal specific features of fPARP1?

Like *h*PARP1/2/3, fPARP1 appears to play a role in the DDR with its catalytic activity directly dependent on the presence of damaged DNA in *A. fumigatus, B. dendrobatidis, M. oryzae*, and *P. anserina* [28,35,37]. Our investigation of the activation mechanism of *Af*-PARP1 showed that, similar to *h*PARP1–3, *Af*-PARP1 undergoes allosteric regulation: in the ground state, residues of the αF helix located in the regulatory HD domain block parts of the active site and prevent NAD^+^ binding. Engagement of damaged DNA by the WGR domain induces conformational changes in the HD domain, opening the active site, and allowing NAD^+^ binding, thus enabling ADP-ribosylation activity. Similar to *h*PARP2/3, the WGR domain of *Af*-PARP1 contains a tyrosine residue (Tyr281) which interacts with 5′-phosphate group at the DNA damage site, making *Af*-PARP1 activation dependent on its presence [28].

While this activation mechanism and phosphate dependency is more reminiscent of *h*PARP2/3, most fPARP1s contain an N-terminal BRCT domain similar to *h*PARP1 (Figure 2A). In *h*PARP1, the BRCT domain can interact with intact DNA and participates in a ‘monkey-bar’ termed chromatin scanning mechanism [65]. However, the BRCT domain is absent from the catalytic multidomain assembly which constitutes the active *h*PARP1 conformation formed upon chromatin lesions recognition [38,66]. Similarly, the *Af*-PARP1 BRCT domain can interact with undamaged DNA and might promote DNA scanning [28]. However, it also appears to form a crucial part of the active *Af*-PARP1:DNA complex and influences both the stability of the complex as well as *Af*-PARP1 activity [28].

## What are the (patho)physiological roles of fungal ADP-ribosylation?

The study of fungal ADP-ribosylation is still in its infancy, but evidence across diverse fungi suggests that it plays a crucial role in virulence and stress adaptation [13,27,28,36,37]. This is in line with its well-established role in bacterial and viral pathogens. In some of these systems, ADP-ribosylation enzymes are injected into host cells to directly manipulate host factors, e.g., the T4 bacteriophage ART Alt and the diphtheria toxin of *Corynebacterium diphtheriae* [67]. Among other functions, this promotes preferential expression of pathogen genes, dampens the host immune response, and improves growth conditions [4,67–69]. In other cases, ADP-ribosylation acts within the pathogen itself. For example, the bacterial rifampin ART can confer resistance by directly modifying and inactivating antibiotics of the rifampicin group commonly used in tuberculosis treatment [70]. Another example is the adaptation of *Staphylococcus aureus* to phagocyte-derived reactive oxygen species (ROS) during infection by utilising SirTM-mediated ADP-ribosylation to protect lipoic acid from oxidation and regulate its transfer to enzyme complexes needed for central metabolism [27,69].

Interestingly, the SirTM-homolog from *C. albicans, Ca*Mfs1 (orf19.2285, IPF9996), is up-regulated in response to oxidative stress in a CAP1-dependent manner [71,72] and was suggested to modulate the fungal oxidative stress response [27]. However, the cellular targets regulated by *Ca*Mfs1, whether ADP-ribosylation, as in bacteria, depend on cross-talk with other PTMs, and whether its mode of action is conserved across other fungi harbouring Mfs1 homologues remains to be elucidated.

Although comparatively little work has been conducted on fungal PARPs, a broad range of (patho)physiological roles for fPARP1, including in morphogenesis, DNA damage repair, telomere function, abiotic stress response, and virulence, have been described [11,13,28–30,34–37]. Whether these represent species-specific roles or are linked to the regulation of common pathway(s) involved in fungal stress response, host adaptation or invasion, remains an exciting open question.

Within the host-microenvironment, fungi are exposed to various stresses, such as phagocyte-derived ROS or antifungal agents, that can either directly or indirectly damage DNA. Therefore, the observations that fPARP1 activity is strictly dependent on the presence of damaged DNA suggest that it is involved in host adaptation by contributing to the DDR. In line with this idea, exposure of *A. nidulans* to camptothecin, a topoisomerase I inhibitor that induces replication-associated double-stranded breaks, increases *parp1* gene expression [73]. Heterozygous deletion of *An*-PARP1 (also termed prpA) resulted in smaller colonies and increased sensitivity to DNA-damaging agents or oxidative stress [30]. However, this response appears to be finely balanced, as fPARP1 overexpression in *P. anaseria* increases sensitivity to phleomycin, paraquat, and farnesol [35]. This is consistent with studies in mice and human HaCaT cells, which suggest that ectopic expression of *h*PARP1 can impair DNA repair and other physiological processes [74,75]. It was suggested that this, at least in part, is due to increased NAD^+^ depletion following PARP activation [76]. Moreover, *h*PARP1 hyperactivation can lead to cell death via the PARthanatos pathways. This involves accumulation of PAR polymers, their translocation into the cytoplasm, interaction and activation of apoptosis-inducing factor (AIF), translocation of AIF to the nucleus, and ultimately cell death [77]. While initially believed to be a caspase-independent process, a recent report suggests that caspase 3 can cleave PARylated *h*PARP1 and that the resulting 89 kDa fragment acts as a PAR chain carrier [78]. Similarly, it was shown that *P. anserina* PARP1 is a substrate for two metacaspases involved in programmed cell death, and fPARP1 was identified as a putative apoptosis-related protein in *Fusarium pseudograminearum* [79,80]. These observations raise the intriguing possibility that fPARP1 may also contribute to fungal apoptosis-like pathways.

In addition to a role in the DDR, fPARP1 also appears to have a role in morphogenesis and conidiation. Overexpression of *An-parp1* resulted in morphological alterations, including formation of fluffy colonies comprised of highly vacuolated aerial hyphae, characteristic of defective conidiation, and reduced spore production [30]. In *M. oryzae*, PARP1 appears to be required for infection-related morphological changes and growth [37]. Deletion of the *Mo-parp1* gene resulted in delayed formation and increased collapse rate of the appressorium, a specialised structure with roles in attachment and invasion of the host, as well as elongated germ tubes. Thus, the reduced virulence of the knockout strains may, in part, result from an impaired ability to form invasive structures and therefore may be a secondary phenotype to a general fitness defect. Moreover, exposure to the DNA synthesis inhibitor hydroxyurea induced PARylation of two 14-3-3 proteins, affecting their ability to dimerise [37]. Dimerisation of these 14-3-3 proteins is required for activation of interacting mitogen-activated protein kinases, which in turn promotes correct appressorium formation. Notably, ectopic expression of *h*PARP1 in *Saccharomyces cerevisiae*, which normally lacks PARP proteins, causes abnormal ADP-ribosylation of essential proteins, which also leads to morphological changes reminiscent of cell cycle mutants and accumulation and stalling of cells in the G_2_/M phase [81–83]. As the growth retardation is reversible via PARP inhibition, the yeast model was utilised as a screening platform for inhibitors of mammalian, plant, and fungal PARPs [84–86].

In *F. oxysporum* f.sp. *niveum*, plants infected with strains lacking *parp1* exhibited delayed disease onset, reduced disease severity, and decreased fungal biomass in infected stems and roots [36]. Unlike *M. oryzae*, this virulence defect is unlikely to relate to fitness or morphology, as deletion of *parp1* from *F. oxysporum* resulted in only a mild reduction in colony diameter on minimal medium, but no other conditions were tested, and it did not exhibit differences in spore length or septa numbers. This reduction in virulence is apparently dependent on the enzymatic activity of *Fon*PARP1, as complementation with the catalytically impaired PARP1 E729K mutant failed to restore WT phenotypes. In humans, the corresponding *h*PARP1 E988K mutation is catalytically impaired (1.25% activity compared with WT), limited to MARylation, and shows increased retention at DNA damage sites [87–89]. IP-MS and yeast two-hybrid assays suggest that *Fon*PARP1 interacts with the Ser/Thr protein kinase *Fon*Kin4, and the authors suggest that phosphorylation of *Fon*PARP1 by *Fon*Kin4 stimulates the former's ADP-ribosylation activity. This is supported by the observation that a *Fon*-*kin4* knockout exhibits similar phenotypes to Δ*parp1* during infection, as well as by previous reports implicating *Fon*Kin4 homologues in fungal morphogenesis and virulence [36,90,91]. While intriguing, questions regarding the interaction remain as Kin4 localises to hyphae septa and macroconidia [36,90,91] whereas *Fon*PARP1 is predominantly nuclear [36]. By contrast, deletion of the PAR-degrading enzyme PARG appears to have no role in pathogenicity, thus suggesting the establishment but not the hydrolysis of ADP-ribosylation is important during *Fusarium* infection [36,42]. However, *parg* appear to have undergone gene duplication as several fungi, including *A. fumigatus*, carry two or more copies [44], hence functional redundancy in the degradation system may explain the lack of phenotype.

In contrast with the pathogenic fungi, in which fPARP1 appears to promote stress tolerance, the fPARP1 homologue of *T. guizhouense*, a beneficial fungal component of plant microbiomes, has been suggested to negatively impact carbon source utilisation under heat stress [34]. Elevated temperatures induce *Tg*PARP1 expression and coincide with an increased NAD^+^ utilisation. Replenishment of NAD^+^ causes depletion of intracellular ATP, affecting energy homeostasis in the ER and a reduction in lignocellulases secretion [34]. This results in reduced lignocellulose utilisation and slower growth under heat stress. Interestingly, overexpression of fPARP1 in *P. anserina* also reduced cellular ATP levels [35].

The reason why *Tg*PARP1 is up-regulated under heat stress and whether it can influence other adaptive responses remains to be elucidated. The beneficial effect of fPARP1 loss on *T. guizhouense*, through limiting NAD^+^ depletion, contrasts with the negative effects on fitness, morphology, or pathogenicity observed in other species (Figure 3) [28,30,34,36,37]. This suggests that the effects of reduced ADP-ribosylation signalling may be species- or context-specific. Further research is, however, needed to fully understand how maintaining a PAR-mediated stress response is balanced against preserving adequate cellular NAD^+^/ATP levels. While many questions remain open, these observations hint at a crucial role of ADP-ribosylation signalling in fungal stress adaptation and virulence. Together with the growing evidence of crucial differences between the mammalian and fungal ADP-ribosylation systems and the success of pharmacologically targeting ADP-ribosylation enzymes in other settings, these findings highlight the potential of fungal ADP-ribosylation signalling as novel antifungal therapy. However, realising this potential will require a better understanding of its (patho)physiological role, the dynamics of signal establishment and removal, its role in cellular energy homeostasis, and the extent to which these pathways are conserved across PARP-carrying fungi.

**Figure 3:**
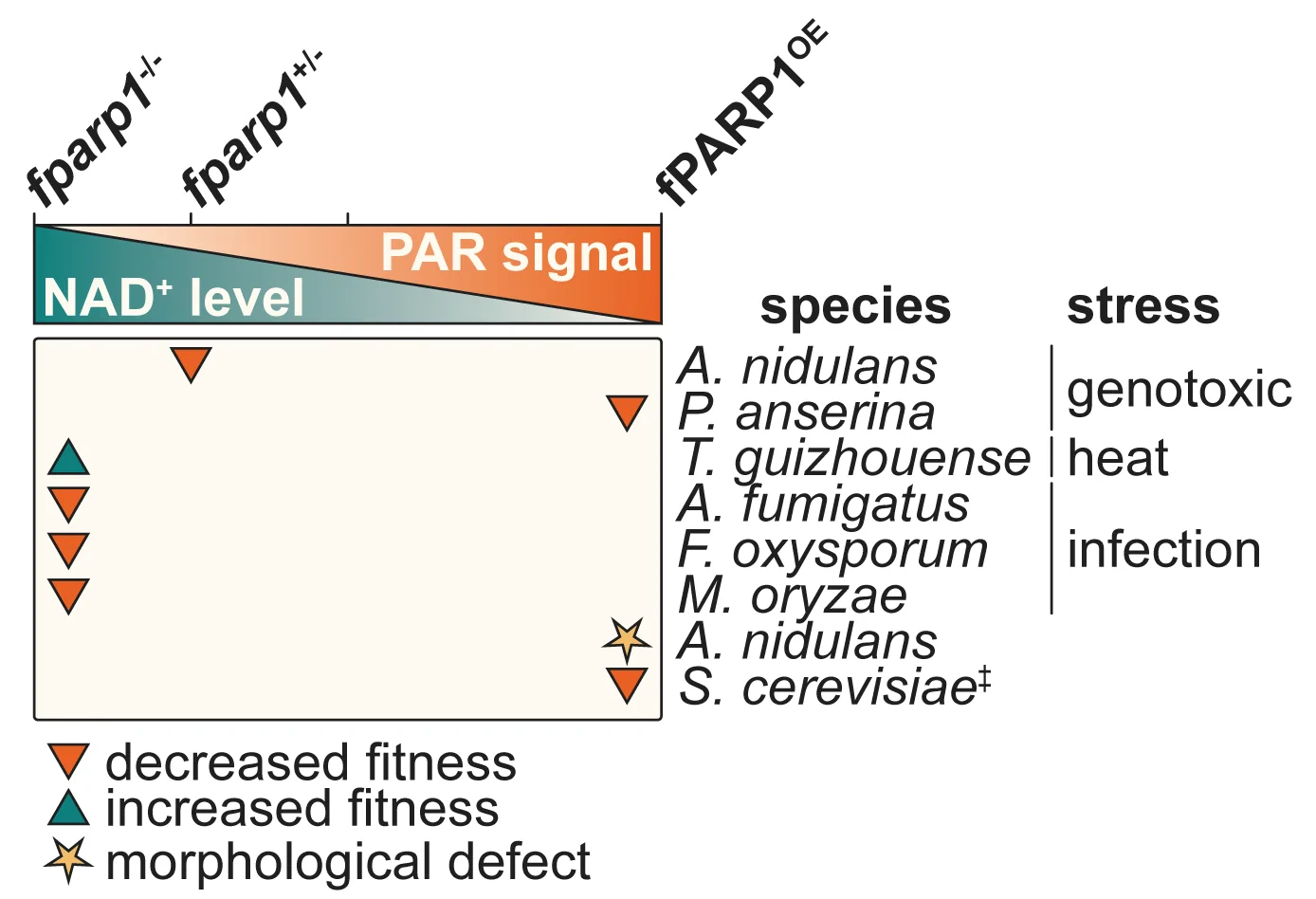
Impact of fPARP1 modulation in different species and contexts Summary of the physiological effects of fPARP1 reduction (homo- and heterozygotic knockout) as well as overexpression in different species under various growth conditions. Together these observations hint at an inverse relationship between PAR signal strength and cellular NAD^+^ content, which may influence fungal fitness in a biphasic manner, with both increased and reduced signalling exerting a negative impact on the organism. ^‡^Effect of ectopic overexpression of PARPs in *S. cerevisiae*, which does not encode an endogenous PARP protein.

## Perspectives

- Fungal ADP-ribosylation is a crucial signalling pathway in stress adaptation as well as a key mechanism supporting virulence of human and plant pathogens.
- Recent advances link ADP-ribosylation to fundamental cellular processes such as DNA repair, morphological switches, and host invasion.
- Many aspects of fungal ADP-ribosylation remain to be revealed: What are the mechanisms by which ADP-ribosylation supports virulence?, What are the dynamics of signal establishment and removal?, and Does ADP-ribosylation regulate common cellular processes or has it been adapted to specific species needs? These are only a few of the questions that beg to be addressed.

## Open Access

Open access for this article is provided via our Subscribe to Open programme, made possible through the support of our subscribing institutions. Learn more here: https://portlandpress.com/pages/open_access_options_and_prices#conditional.

## Abbreviations

*Af*-PARP1: *A. fumigatus* PARP1
AIF: apoptosis-inducing factor
ARHs: (ADP-ribosyl)hydrolases
ART: (ADP-ribosyl)transferase
ARTCs: cholera toxin-like ARTs
DDR: DNA damage response
D-loop: donor loop
fPARP1: fungal PARP1
fPARP2: fungal PARP2
HE: helical extension
HPF1: histone PARylation factor 1
*h*PARP1: human PARP1
Mfs1: macrodomain-fused SirTM 1
NADARs: ‘NAD- and ADP-ribose’-associated enzymes
PAE: predicted aligned error
PTMs: posttranslational modifications
ROS: reactive oxygen species
